# Functional and structural connectivity of the subregions of the amygdala in ADHD children with or without ODD

**DOI:** 10.1186/s12888-025-06500-4

**Published:** 2025-01-24

**Authors:** Zhao-Min Wu, Peng Wang, Xue-Chun Liu, Qing-Chao Zhou, Xiao-Lan Cao, Li Sun, Lu Liu, Qing-Jiu Cao, Li Yang, Yu-Feng Wang, Ying Qian, Bin-Rang Yang

**Affiliations:** 1 Shenzhen Children’s Hospital, Shenzhen, 518000 China; 2 Affiliated Shenzhen Children’s Hospital of Shantou University Medical College, Shenzhen, 518000 China; 3 Cardiac Rehabilitation Center, Fuwai Hospital, CAMS & PUMC, Beijing, 100037 China; 4 Institute of Mental Health, Peking University Sixth Hospital, National Clinical Research Center for Mental Disorders (Peking University Sixth Hospital), Beijing, 100191 China; 5 Key Laboratory of Mental Health, Ministry of Health, Peking University, Beijing, 100191 China

**Keywords:** ADHD, ODD, Amygdala, Functional connectivity, Structural connectivity

## Abstract

**Objectives:**

The current study aimed to investigate the structural and functional connectivity of the subregions of the amygdala in children with Attention Deficit/Hyperactivity Disorder (ADHD) only or comorbid with Oppositional Defiant Disorder (ODD).

**Methods:**

A total of 354 children with ADHD-only, 161 children with ADHD and ODD (ADHD + ODD), and 100 healthy controls were enrolled. The Child Behavior Checklist (CBCL) and the Behavior Rating Inventory of Executive Function (BRIEF) were filled out by caregivers. Analysis of covariance (ANCOVA) was performed to test group-wise differences in these behavioral measures. A subsample comprising 209 participants underwent a resting-state functional MRI scan and a diffusion-weighted imaging (DWI) scan. Functional connectivity and structural connectivity were calculated using bilateral subregions of the Amygdala as seeds. Between-group voxel-wise comparisons were conducted.

**Results:**

The ADHD + ODD group had more anxious/depressed moods, more delinquent and aggressive behaviors, more emotional control problems, and more inhibition deficits than the ADHD-only group (all *P*_Bonferroni−corrected_ < 0.05). Compared with the control and ADHD + ODD groups, the ADHD-only group displayed increased FC strength between the amygdala subregions and the left caudate, left putamen, and frontal cortex. Regarding structural connectivity (SC), the ADHD-only group demonstrated higher streamline density in the left internal capsule, corpus callosum, and the right superior corona radiata. The altered SC was associated with emotional problems in children with ADHD, while the altered FC was associated with other ADHD-related clinical features.

**Conclusions:**

Altered structural and functional connectivity of the subregions of the amygdala in children with ADHD compared with their healthy counterparts were respectively associated with ADHD-related behavioral and emotional problems.

**Clinical trial number:**

: not applicable.

**Supplementary Information:**

The online version contains supplementary material available at 10.1186/s12888-025-06500-4.

## Introduction

Attention Deficit Hyperactivity Disorder (ADHD) and Oppositional Defiant Disorder (ODD) often cooccur in children and adolescents. About 60% of ADHD patients were also diagnosed as having ODD [1]. According to the Diagnostic and Statistical Manual of Mental Disorders (DSM-5), ADHD is characterized by inattention and/or hyperactivity/impulsivity, and ODD is defined by irritable and angry moods and defiant and disobedient behaviors. Comorbid ODD in individuals with ADHD was found to be related to more functional impairment and a worse long-term prognosis [2–5].

Several previous studies have shown that occurring ODD was linked to a higher level of emotional liability and delinquent behaviors in children with ADHD [3, 6]. A previous study has demonstrated dimensional correlations between cognitive inhibition and visuospatial episodic memory and ADHD symptoms and between cognitive flexibility and ODD symptoms in a sample referred to clinical evaluation for cognitive and/or socio-emotional problems [7].

As a neurodevelopmental disorder, magnetic resonance imaging (MRI) was often utilized to explore the underlying neural correlates of ADHD. MRI, as a non-invasive technique, provides information about the functional and structural characteristics of brain circuits, allowing in-depth exploration of mechanisms of neurodevelopmental disorders [8]. Previous studies have demonstrated both structural and function alterations in multiple brain regions in subjects with ADHD or ODD compared to healthy controls [9, 10]. Regarding ADHD comorbid ODD, most of the existing studies have focused on the structural abnormalities. A study comprising 92 participants revealed that boys with ADHD + ODD/CD had a thicker cortex than the ADHD-only group and the healthy controls in a right rostral middle frontal cluster, which was also shown to be related to the ODD/CD symptoms, even when controlling for ADHD symptoms [11]. Another recent study, which included only 81 participants, identified altered gray matter volume and functional connectivity in the cerebellum [12]. One large sample size study (*n* = 543) showed that ADHD + ODD was uniquely associated with volumetric reductions in several structures (e.g., the precuneus and the medial orbitofrontal cortex) [13]. In terms of white matter microstructure, comorbid ODD in ADHD was associated with lower fractional anisotropy (FA) (an index derived from the diffusion MRI data, higher FA was found to be related to higher white matter axonal integrity and organization [14]) in left frontotemporal white matter, which was related to antisocial behaviors in patients with ADHD and ODD (ADHD + ODD) [15]. Nevertheless, in another sample consisting of subjects with ADHD, disruptive mood dysregulation disorder, and healthy individuals, there are no specific white matter microstructural underpinnings of inattention or irritability symptoms [16]. This inconsistency indicated that the irritable mood and aggressive behaviors might have different neural correlates.

As mentioned, angry and irritable moods, as well as defiant and disobedient behaviors, characterized the core features of ODD. In the human brain, several regions are responsible for the rise of emotions, including the amygdala [17]. In particular, the interplay between the amygdala and the prefrontal cortex plays a vital role in human emotion-generative and regulatory systems [18, 19]. Emotional dysregulation symptoms in ADHD were also found to be associated with structural abnormalities in the amygdala [20]. The amygdala can be divided into several subregions with functional similarities [21]. Previously, altered functional connectivity of different subregions of the amygdala was shown to be associated with several psychiatric disorders, e.g., obsessive-compulsive disorder (OCD) [22]. A previous study showed that the functional connectivity between basolateral amygdala and other cortical regions was sensitive to fear stimuli [23]. In children with ADHD, the right superficial amygdala exhibited significantly higher dynamic functional connectivity with the right prefrontal cortex in children in the ADHD group compared with the control group [24]. The abnormal resting-state functional connectivity between the superficial amygdala, the dorsolateral prefrontal cortex, and the inferior parietal lobe was related to emotional dysregulation symptoms in children with ADHD [25].

In summary, ADHD and ODD often co-exist, and emotional dysregulation and impulsive behavior might bridge the two disorders. Results from previous studies have highlighted the critical role of the amygdala in emotional regulation in typical developing children and individuals with neurodevelopmental disorders like ADHD. Nevertheless, few studies have investigated whether the abnormal structure and function of the amygdala underlies the cooccurrence of ADHD and ODD. In addition, how the emotional and behavioral symptoms were related to the functional and structural alterations in the amygdala also remains unclear. Therefore, the current study aimed to investigate the functional and structural connectivity of the amygdala subregions in a clinical sample comprising a group of ADHD individuals with or without ODD and healthy controls. Our multimodal database, with multiple dimensional behavioral and emotional measures and multimodal MRI scans, would provide insights into the neural correlates of the comorbidity between ADHD and ODD.

## Methods

*Participants*: The current study included a clinical sample of 354 children with ADHD-only (no other comorbidity) and 161 children with ADHD and ODD (all aged 6–15) recruited from Shenzhen Children’s Hospital and 100 healthy controls recruited from schools in Shenzhen. A subsample comprising 90 children with ADHD-only, 47 children with ADHD and ODD, and 72 healthy individuals (all right-hand dominant) went through a resting-state functional MRI scan and a diffusion-weighted imaging (DWI) scan. All the ADHD patients visited the Child psychiatrists due to “having difficulties in sustaining their attention at study,” and the diagnosis of ADHD and/or ODD was first made at the outpatient clinic after initial clinical interviews and evaluations. Besides, all participants and their parents were interviewed by a clinician using the Schedule for Affective Disorders and Schizophrenia for School-Age Children-Present and Lifetime version (K-SADS-PL) [26] to confirm or rule out any psychiatric diagnosis. The parent who knew the child best was regarded as the primary informant. The inclusion criteria for the ADHD and the control groups can be found in the supplementary material. This work was approved by the Ethics Committee of Shenzhen Children’s Hospital, following the criteria set by the Declaration of Helsinki (identification number: 201701805). Informed consent was obtained from parents of children before the study.

*Clinical Assessments*: The ADHD rating scale was filled by the parents, providing information about the severity of the ADHD core symptoms. The Child Behavior Checklist (CBCL) was utilized to assess all participants’ behavioral and emotional problems. The CBCL items can be summarized into eight factors: withdrawn, somatic complaints, anxiety/depression, social problem, thought problem, attention problem, delinquent behavior, and aggressive behavior. In addition, the Behavior Rating Inventory of Executive Function (BRIEF) was used to assess everyday executive function in children. The BRIEF scale can be divided into eight subscales: inhibit, working memory, planning, monitor, initiate, shift, organization of materials, and emotional control [27].

*Cognitive Assessments*: The Wechsler Intelligence Scale For Children: Fourth Edition (WISC-IV) was utilized to systematically assess each individual’s intelligence quotient (IQ). It generates five indexes: the full-scale IQ (FSIQ), the Verbal Comprehension Index (VCI) Composite Score, the Perceptual Reasoning Index (PRI) Composite Score, the Working Memory Index (WMI) Composite Score, and the Processing Speed Index (PSI) Composite Score.

### Brain imaging acquisition

Multimodal MRI scans (T1 weighted, diffusion-weighted, and resting-state function MRI) were acquired on the same 3T Siemens Skyra scanner with a standard 12-channel head coil in Shenzhen Children’s Hospital. Parameters can be found in the supplementary material.

*Preprocessing and processing of MRI Images*: All the RS-fMRI images were preprocessed using FSL (http://fsl.fmrib.ox.ac.uk/fsl) and python (https://www.python.org/) with the independent Components Analysis-based Automatic Removal Of Motion Artifacts (ICA-AROMA) technique applied to remove the head motion artifacts [28]. The timeseries of each subregion and the functional connectivity maps were built using dual regression [29]. Diffusion-weighted images were also processed using FSL. The probabilistic tractography was performed, using subregions of the Amygdala as seeds, resulting in streamline density maps for each individual. The streamline density map contains for each voxel a count of how many of the streamlines intersected with that voxel. Streamline density is one measure that has been proposed to reflect structural connectivity strength [30]. Between-group differences were estimated by performing voxel-wise comparisons on these streamline density maps. All the voxel-wise comparisons, whether of the DWI or fMRI, were conducted using the Randomise algorithm in FSL [31]. Cluster-wise statistics corrections were performed by using TFCE (Threshold-Free Cluster Enhancement), providing p-values corrected for family-wise error (FWE) [32]. To balance the false positives and the false negatives, only those significant clusters larger than ten voxels were reported [33, 34]. More details about the preprocessing of the MRI images can be found in the supplementary material.

*Statistical Analysis*: To estimate the group-wise differences in the behavioral measures, Analysis of covariance (ANCOVA) was performed, with age and sex as covariates. Bonferroni correction was applied to correct for multiple comparisons. Since there are eight factors for the CBCL and BRIEF scales, the significant p-value level was set to 0.05/8 = 0.00625 for these scales’ measures. The significant p-value for WISC-IV measures was then set to 0.05/5 = 0.01; for the ADHD-RS, the number was set to 0.05/2 = 0.025. In exploring the brain-behavior correlation, the mean functional connectivity strength and the mean streamline density of the clusters showing between-group differences were extracted. Regression models were built with behavioral indicators as the dependent variable, functional or structural measures as an independent variable, and age and sex as covariates. FDR correction was used to correct for multiple comparisons, considering the interrelationship between brain and behavioral measures.

## Results

### Demographic characteristics

In the whole sample, both ADHD groups are younger than the control group (both *P* < 0.0001). Nevertheless, in the sub-sample, those subjects with MRI scales, there are no significant differences between the ADHD and the control groups in terms of age. In addition, there are more male subjects in both ADHD groups compared with the control group (both *P* < 0.0001). In the sub-sample, the ratio of male subjects was higher in the ADHD-only group but not in the ADHD + ODD group, compared with the controls. As expected, after controlling for age and sex, both ADHD groups scored higher on the ADHD rating scale and had lower IQ indexes in the WISC-IV compared with the control group (all *P*_*Bonferroni−corrected*_ <0.05). Details are summarized in Table 1.

**Table 1:** Demographic information of the ADHD and the control groups

	ADHD-only	ADHD + ODD	Control	F / χ2	*P* value	A1 vs. C	A2 vs. C	A1 vs. A2
Sample Size	354	161	100					
Age	8.21 ± 1.71	8.45 ± 1.69	9.35 ± 1.37	18.49	< 0.0001	-6.08 (< 0.0001) *	-4.29 (< 0.0001) *	1.50 (0.29)
Sex (Male)	301 (85.03%)	138 (85.71%)	55(55.00%)	48.5	< 0.0001	41.54 (< 0.0001) *	30.21 (< 0.0001) *	0.041 (0.84)
Sub-Sample	90	47	72					
Sub-Age	8.88 ± 1.35	9.26 ± 1.59	9.35 ± 1.38	2.46	0.088			
Sub-Sex (Male)	69 (76.67%)	35 (74.47%)	42 (58.33%)	7.00	0.030	6.23 (0.013) *	3.24 (0.072)	0.082 (0.78)
Inattention	16.56 ± 5.24	16.90 ± 5.39	7.24 ± 6.23	81.19	< 0.0001	12.02 (< 0.0001) *	10.92 (< 0.0001) *	0.55 (0.85)
Hyperactivity/Impulsivity	12.97 ± 5.84	14.35 ± 5.67	5.05 ± 5.33	47.82	< 0.0001	8.44 (< 0.0001) *	9.18 (< 0.0001) *	2.30 (0.056)
Full-Scale IQ	93.92 ± 10.74	95.87 ± 10.95	104.55 ± 9.17	32.69	< 0.0001	-8.08 (< 0.0001) *	-5.97 (< 0.0001) *	1.91 (0.13)
VCI	95.66 ± 11.61	97.72 ± 11.24	102.33 ± 10.25	14.95	< 0.0001	-5.44 (< 0.0001) *	-3.51 (0.0013) *	1.99 (0.11)
PRI	101.21 ± 11.80	101.83 ± 12.68	106.60 ± 10.51	7.20	0.00081	-3.76 (0.00055) *	-3.06 (0.0064) *	0.50 (0.87)
WMI	89.03 ± 10.37	89.95 ± 10.94	97.56 ± 10.03	19.05	< 0.0001	-6.13 (< 0.0001) *	-4.93 (< 0.0001) *	0.88 (0.65)
PSI	93.02 ± 12.01	95.44 ± 10.84	106.82 ± 11.24	39.16	< 0.0001	-8.85 (< 0.0001)	-6.54 (< 0.0001)	2.08 (0.092)

Abbreviations: A1 represents the ADHD-only group, while A2 represents the ADHD + ODD group. * Indicates a Bonferroni-corrected *P* value below 0.05. VCI = Verbal Comprehension Index; PRI = Perceptual Reasoning Index; WMI = Working Memory Index; PSI = Processing Speed Index; FSIQ = full-scale intelligence quotient;

### Clinical features and daily-life executive function of the ADHD and control groups

As expected, after controlling for age and sex, both ADHD groups scored higher on each CBCL and BRIEF subscale (all *P*_*Bonferroni−corrected*_ <0.05), except for the somatic subscale of CBCL. The ADHD + ODD group scored higher than the ADHD-only group in several subscales from CBCL, e.g., delinquent behavior and aggressive behavior, and BRIEF, e.g., inhibit and emotional control (all *P*_*Bonferroni−corrected*_ <0.05). In addition, the ADHD + ODD group had a marginally significantly higher level of anxious/depressed mood compared with the ADHD-only group, according to CBCL (P_norminal_ = 0.011). Details are summarized in Table 2.

**Table 2:** Clinical features and daily-life executive function of the ADHD and control groups

	ADHD-only	ADHD + ODD	Control	F	*P*	A1 vs. C	A2 vs. C	A1 vs. A2
withdrawn	2.94 ± 2.65	3.06 ± 2.27	1.44 ± 1.77	8.87	0.00016	4.02 (0.00019) *	3.99 (0.00021) *	0.38 (0.92)
Somatic complaints	1.61 ± 1.96	1.39 ± 1.75	0.74 ± 1.54	4.12	0.017	2.82 (0.013)	1.92 (0.13)	-1.13 (0.49)
Anxiety/depression	3.73 ± 3.63	4.82 ± 3.86	1.35 ± 2.43	16.62	< 0.0001	4.28 (< 0.0001) *	5.73 (< 0.0001) *	2.87 (0.011)
Social problem	4.07 ± 2.78	4.42 ± 2.71	1.37 ± 1.55	22.68	< 0.0001	6.23 (< 0.0001) *	6.57 (< 0.0001) *	1.20 (0.44)
Thought problem	1.13 ± 1.45	1.26 ± 1.54	0.37 ± 1.14	6.68	0.0014	3.34 (0.0024) *	3.58 (0.0010) *	0.75 (0.73)
Attention problem	8.21 ± 3.40	8.77 ± 3.21	1.74 ± 2.17	89.71	< 0.0001	12.71 (< 0.0001) *	12.77 (< 0.0001) *	1.52 (0.27)
Delinquent behavior	3.78 ± 2.87	4.66 ± 3.05	1.49 ± 2.14	20.48	< 0.0001	4.92 (< 0.0001) *	6.38 (< 0.0001) *	2.96 (0.0085) *
Aggressive behavior	10.74 ± 6.47	14.80 ± 6.74	3.75 ± 3.85	54.42	< 0.0001	6.72 (< 0.0001) *	10.14 (< 0.0001) *	6.35 (< 0.0001) *
Inhibit	18.29 ± 4.71	19.80 ± 4.95	11.95 ± 1.90	67.24	< 0.0001	9.66 (< 0.0001) *	11.04 (< 0.0001) *	2.99 (0.0084) *
Shift	12.19 ± 2.79	12.45 ± 2.61	9.91 ± 1.86	27.90	< 0.0001	6.80 (< 0.0001) *	6.63 (< 0.0001) *	0.82 (0.69)
Initiate	15.02 ± 3.02	15.12 ± 3.24	10.69 ± 2.26	73.40	< 0.0001	11.47 (< 0.0001) *	10.21 (< 0.0001) *	0.18 (0.98)
Emotional Control	15.12 ± 4.13	18.22 ± 4.30	11.88 ± 2.35	55.93	< 0.0001	5.77 (< 0.0001) *	10.53 (< 0.0001) *	6.59 (< 0.0001) *
Working Memory	22.04 ± 3.90	21.89 ± 3.61	14.07 ± 3.01	139.61	< 0.0001	16.00 (< 0.0001) *	13.72 (< 0.0001) *	-0.38 (0.92)
Planning	25.36 ± 4.78	26.78 ± 4.30	17.74 ± 4.15	105.98	< 0.0001	12.91 (< 0.0001) *	13.25 (< 0.0001) *	2.22 (0.069)
Organization of Material	13.15 ± 3.16	13.40 ± 2.81	9.48 ± 2.59	51.78	< 0.0001	9.46 (< 0.0001) *	8.89 (< 0.0001) *	0.64 (0.80)
Monitor	18.70 ± 3.17	19.44 ± 2.82	12.99 ± 2.92	106.50	< 0.0001	13.16 (< 0.0001) *	13.20 (< 0.0001) *	1.83 (0.16)

Abbreviations: A1 represents the ADHD-only group, while A2 represents the ADHD + ODD group. * Indicates a Bonferroni-corrected *P* value below 0.05

### Functional and structural connectivity of the amygdala

**Fig. 1:**
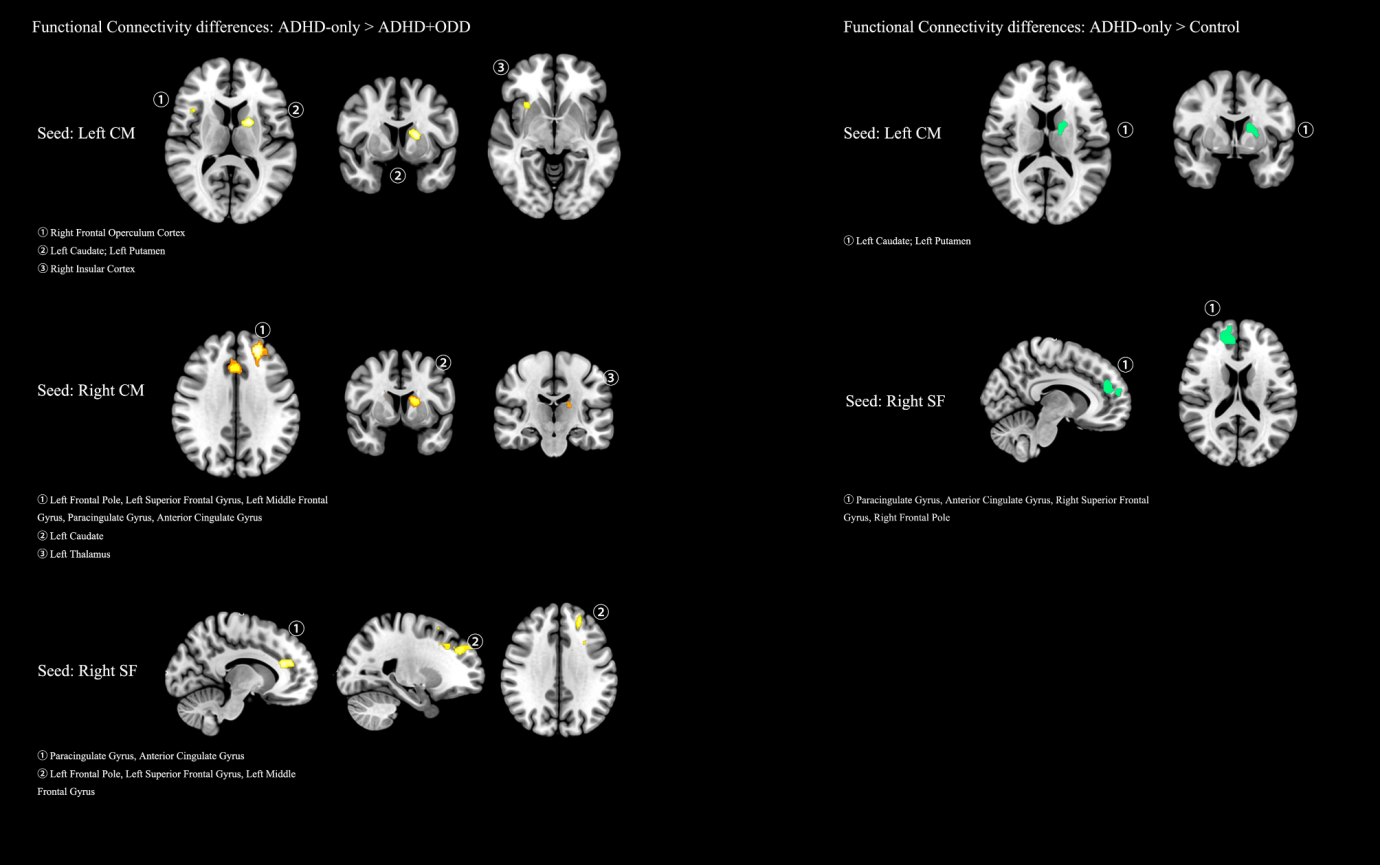
The spatial maps of the clusters showing significant between-group functional connectivity differences displayed on an MNI_T1_1mm_brain. Seed regions: CM = the cortical/medial (CM) part of the Amygdala; BL = the basolateral (BL) part of the Amygdala; SF = the superficial (SF) part of the Amygdala

**Fig. 2:**
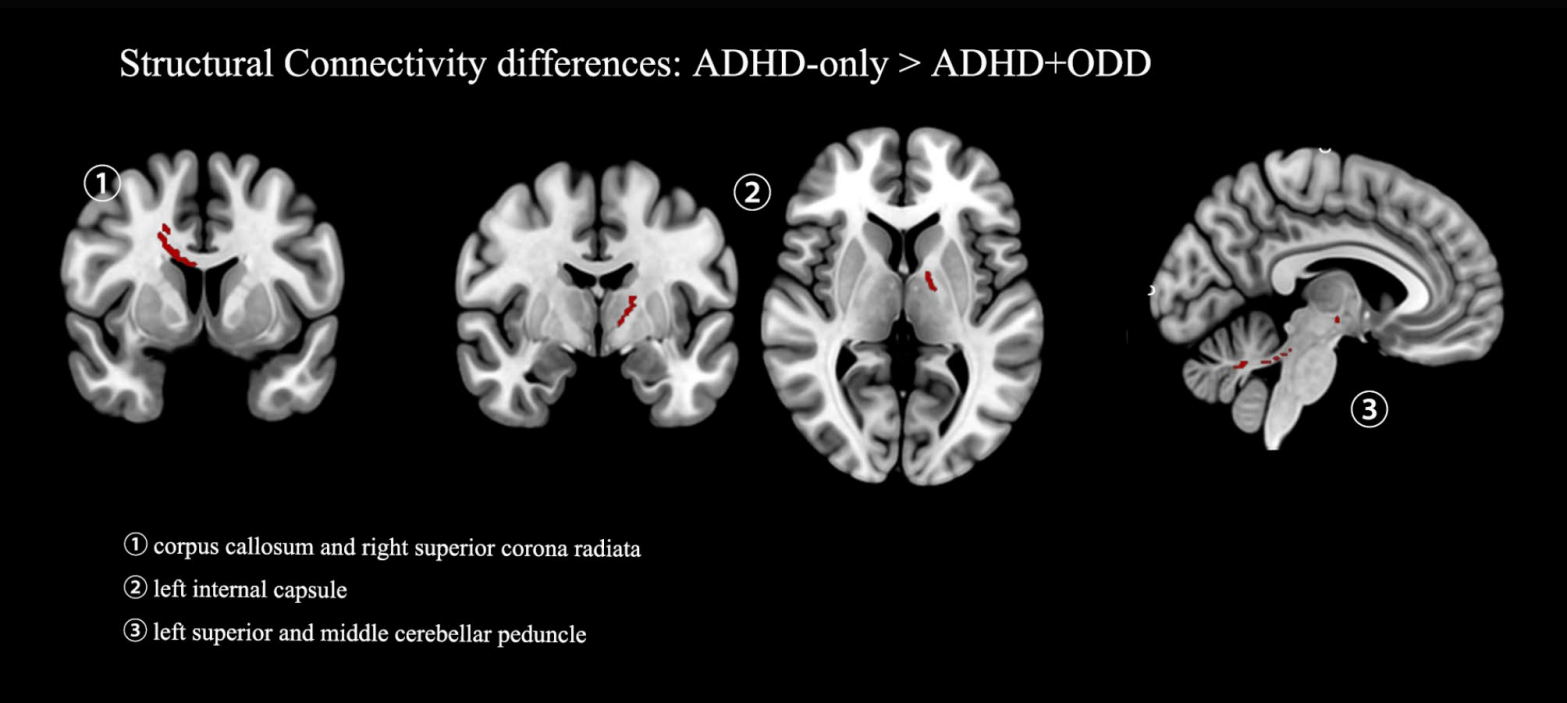
The spatial maps of the clusters showing significant between-group structural connectivity differences displayed on an MNI_T1_1mm_brain. Seed regions: CM = the cortical/medial (CM) part of the Amygdala; BL = the basolateral (BL) part of the Amygdala; SF = the superficial (SF) part of the Amygdala

Compared with the control group and the ADHD + ODD group, the ADHD-only group displayed elevated functional connectivity between the left cortical/medial (CM) subregion of the amygdala and the left caudate and putamen. Additionally, the ADHD-only group had stronger functional connectivity than the control group between the right superficial (SF) part of the amygdala and the right frontal regions, e.g., paracingulate gyrus, anterior cingulate gyrus, right superior frontal gyrus, and right frontal pole. Compared with the ADHD + ODD group, the ADHD-only group also presented increased function connectivity between the left cortical/medial (CM) subregion of the amygdala and the right insular cortex, and the right frontal operculum cortex, between the right cortical/medial (CM) subregion of the amygdala and left frontal regions, left caudate, and left thalamus, as well as between the right superficial (SF) part of the amygdala and the left frontal regions. Alterations in regional streamline density were observed in the corpus callosum, left internal capsule, left superior cerebellar peduncle, and middle cerebellar peduncle when comparing the ADHD-only and the ADHD + ODD groups. There is no significant difference identified between the ADHD-only or the ADHD + ODD group and the control group. Details are summarized in Table 3; Fig. 1, and Fig. 2.

**Table 3:** Voxel-wise comparisons of functional and structural connectivity of subregions in the Amygdala of the ADHD and the control groups

	Seed region	comparisons	Cluster	Voxels	coordinates	Regions
Functional connectivity	Left CM	A1 > A2	1	129	-20 4 12	Left Caudate; Left Putamen;
			2	37	28 16 − 6	Right Insular Cortex;
			3	15	42 16 10	Right Frontal Operculum Cortex;
	Left CM	A1 > C	1	148	-22 2 10	Left Caudate; Left Putamen;
Functional connectivity	Right CM	A1 > A2	1	1573	-24 44 34	Left Frontal Pole, Left Superior Frontal Gyrus, Left Middle Frontal Gyrus, Paracingulate Gyrus, Anterior Cingulate Gyrus;
			2	173	-18 4 14	Left Caudate
			3	24	-18 -20 14	Left Thalamus;
Functional connectivity	Right SF	A1 > A2	1	266	-14 42 20	Left Superior Frontal Gyrus, Paracingulate Gyrus, Anterior Cingulate Gyrus;
			2	195	-22 40 30	Left Frontal Pole, Left Superior Frontal Gyrus, Left Middle Frontal Gyrus;
			3	55	-4 28 42	Left Superior Frontal Gyrus, Paracingulate Gyrus;
			4	50	-26 0 40	Left Middle Frontal Gyrus;
			5	26	6 20 20	Anterior Cingulate Gyrus;
			6	12	-14 1030	Anterior Cingulate Gyrus;
Functional connectivity	Right SF	A1 > C	1	484	14 48 20	Paracingulate Gyrus; Anterior Cingulate Gyrus; Right Superior Frontal Gyrus, Right Frontal Pole;
Structural connectivity	Left SF	A1 < A2	1	69	-10 -8 -2	Left Internal Capsule;
			2	60	22 10 34	Body of Corpus Callosum, Right Superior corona radiata;
			3	56	-2 -32 -18	Left Superior cerebellar peduncle;
			4	29	-24 -48 -36	Middle cerebellar peduncle;

Abbreviations: A1 represents the ADHD-only group, while A2 represents the ADHD + ODD group. * Indicates a Bonferroni-corrected *P* value below 0.05. Seed regions: CM = the cortical/medial (CM) part of Amygdala; BL = the basolateral (BL) part of Amygdala; SF = the superficial (SF) part of Amygdala

### Brain-behavior relationships

Brain-behavior association tests revealed that the functional connectivity between the left cortical/medial (CM) subregion of the amygdala and other subcortical regions were significantly associated with several measures of the BRIEF scales, including shift, working memory, and monitor (all *P*_*fdr*_ <0.05). In addition, significant associations were identified between the structural connectivity strength of the corpus callosum and the withdrawn and anxiety/depression measures from CBCL (both *P*_*fdr*_ <0.05), as well as between the structural connectivity strength of the middle cerebellar peduncle and the thought problems and delinquent behaviors (both *P*_*fdr*_ <0.05). Details are summarized in supplementary materials from sTable 1 to sTable 4.

## Discussion

The current study examined the clinical features of children with ADHD and ODD. The results showed that both ADHD groups had more emotional and behavioral problems, and they were more impaired in daily-life executive functions compared with the healthy controls. Additionally, compared with the ADHD-only group, the ADHD + ODD group had more anxiety/depression mood, more delinquent and aggressive behaviors, and more impairment in inhibition and emotional control abilities in daily life. Regarding brain function, the ADHD group displayed increased functional connectivity of the bilateral centromedial amygdala and the right superficial amygdala, compared to the control group and the ADHD + ODD group. In addition, lower white matter connection density of the left superficial amygdala was identified in the ADHD group compared with the ADHD + ODD group in the corpus callosum, right corona radiata, left internal capsule, and cerebellar peduncle. Brain-behavior correlation revealed that the altered functional connectivity was associated with ADHD-related behavioral problems, and the altered structural connectivity was associated with ODD-related emotional regulation problems.

ODD is common in children and adolescents with ADHD. As expected, increased levels of delinquent and aggressive behaviors were present in the ADHD + ODD group, compared to the ADHD-only group [35]. In addition, the CBCL anxiety/depression score was also nominally significantly higher in the ADHD + ODD group compared with the ADHD-only group. Although ODD is characterized by “angry/irritable” moods, previous studies have also demonstrated close relationships between anxiety mood and ODD symptoms [36, 37]. Regarding the daily-life executive function, the ADHD + ODD group had more impaired inhibition and emotional control abilities than the ADHD-only group. The inhibit subscale of the BRIEF includes multiple items related to impulsive behaviors, which were closely related to ODD symptoms [38, 39]. The emotional control subscale includes items related to irritability, anger, and emotional lability, which were part of the core symptoms of ODD [40]. These results were consistent with those of a previous study [41]. A previous systematic review and meta-analysis demonstrated that subjects with disruptive behavior disorders, including ODD, were more impaired than the control group in behavioral inhibition, cognitive inhibition, and other executive function domains [42]. Compared with ADHD-only, subjects with ADHD + ODD were also found to have more impaired response inhibition [43, 44]. Our results, along with results from all these previous studies, have implicated that impulsivity and related symptoms might be the key symptoms linking ADHD to ODD, which should be carefully managed through pharmacological or non-pharmacological treatments to prevent a bad long-term prognosis.

In a longitudinal general population-based cohort, functional connectivity (FC) between the prefrontal cortex (PFC) and the amygdala was found to decrease with age, and the stronger PFC-amygdala functional connectivity was related to lower mood variability during adolescence [45]. The Amygdala is a complex structure. In mice, the basal and lateral amygdala have different neurons, leading to different functions [46]. The basolateral amygdala (BLA) is thought to contribute to the flexibility in behavioral outcomes [47]. A previous study using an accelerated cohort longitudinal design (aged 10–25 years) revealed that FC between the centromedial amygdala (CMA) and prefrontal cortex decreased from late childhood, and the FC between the CMA–rostral anterior cingulate cortex is positively associated with anxiety symptoms during early adulthood [48]. The abnormal CMA-frontal cortex was also found to be associated with family cohesion and expressiveness [49], which is related to the rise of ODD symptoms [50]. In addition, childhood maltreatment experience was negatively associated with FC between the right superficial amygdala (SFA) and the anterior cingulate cortex and postcentral gyrus [51]. In young children with ADHD, hypo-connectivity between different parts of the cortex and left putamen/pallidum/amygdala was identified in the meta-analysis [52]. Regarding emotional dysregulation symptoms in ADHD, heterogeneous relationships were observed between the functional connectivity of the amygdala and emotional symptoms [20]. For instance, Yu et al. demonstrated that the FC between subregions of the amygdala and cortical regions were altered in children with ADHD compared with healthy controls, and the reduced negative FC of SFA with the dorsolateral prefrontal cortex and inferior parietal lobe was related to higher emotional lability (EL) symptoms in boys with ADHD [25]. In another study, the EL symptoms were positively associated with FC between the amygdala and rostral anterior cingulate cortex [53]. In a mixed group of children with disruptive mood dysregulation disorder, anxiety disorder, and/or attention-deficit/hyperactivity disorder and healthy youth, the decreased amygdala connectivity to the cingulate, precentral gyrus, and thalamus was shown to be associated with higher anxiety. In contrast, higher irritability was related to increased neural activity in several brain regions but not with the amygdala connectivity [54]. The current study observed higher FC in children with ADHD-only between bilateral CMA and right SFA, and frontal regions and other subcortical regions, compared to children with ADHD + ODD and the healthy controls. Nevertheless, we did not observe any significant correlation between the aberrant FC and emotional symptoms. Instead, significant associations were identified between the altered FC and several indexes from BRIEF, including shift, working memory, and monitor, which were previously reported to be impaired in subjects with ADHD [55].

A mega-analysis of whole brain microstructural integrity (indicated by fractional anisotropy (FA)) revealed that the FA values of the right cingulum, the right superior longitudinal fasciculus, and the left uncinate fasciculus were nominally negatively associated with withdrawn (depressed) mood, thought problems, and aggressive behaviors respectively in population-based cohorts [56]. Studies of a community cohort of children also observed that the FA values of the cingulum-callosal regions were associated with the CBCL Anxiety/Depression and Emotional Dysregulation scores [57, 58]. A longitudinal study showed that structural connectivity between the CMA and rostral anterior cingulate cortex and PFC decreased with age and is positively associated with anxiety during late childhood [48]. In terms of the ADHD population, several white matter pathways, including the generalized FA value of the corpus callosum, were previously found to be negatively associated with the emotional dysregulation symptom severity in children with ADHD [59]. The current study also observed that the white matter structural connectivity strength was associated with emotional and ODD symptoms in ADHD. In particular, the left internal capsule consists of projection fibers to and from the cortex, and the caudal end of the internal capsule forms the cerebral peduncle. The internal capsule connects the prefrontal cortex and the basal amygdala [60], responsible for emotional control [61]. Alterations in the functional and structural concavity of the sub-regions of the amygdala associated with ADHD core symptoms and emotional dysregulation traits indicated that the amygdala might be a candidate target for future Transcranial Magnetic Stimulation (TMS) in ADHD subjects [62].

Limitations: The current study has explored the functional and structural connectivity alterations of subregions of the amygdala in a clinical cohort of ADHD patients with or without ODD and a group of healthy controls. There are more male subjects in the ADHD group than in the control group. Future studies with more female individuals are warranted. In addition, our sample is a single-site sample, and the generalization of the conclusion might be limited to the population with similar cultural backgrounds. Tractography might be biased towards easy-to-track pathways, and future studies incorporating other white matter microstructural metrics might provide alternative perspectives.

In summary, comorbid ODD in ADHD is associated with more anxiety/depression moods. Compared with the control group and the ADHD + ODD group, the ADHD-only group displayed increased functional connectivity between the left cortical/medial (CM) subregion of the amygdala and the left caudate and putamen, which were associated the daily-life executive function deficits in ADHD. Differences in regional streamline density were observed in the corpus callosum, left internal capsule, left superior cerebellar peduncle, and middle cerebellar peduncle between the ADHD-only and the ADHD + ODD groups, which was associated dimensionally with emotional and delinquent symptoms in ADHD.

## Electronic supplementary material

Below is the link to the electronic supplementary material.

Supplementary Material 1

## Data Availability

Data are available from the corresponding author (Prof. Dr. Bin-Rang Yang) upon reasonable request。.
