# Supplementary material for: Functional and structural connectivity of the subregions of the amygdala in ADHD children with or without ODD
Source: BMC Psychiatry. 2025 Jan 24;25:74. doi: 10.1186/s12888-025-06500-4 (PMC11763135; doi:10.1186/s12888-025-06500-4)
Supplement: Supplementary file 1 — Supplementary Material 1 [file 12888_2025_6500_MOESM1_ESM.docx]

# Supplementary material

Supplementary Methods

**Participants**

1. inclusion criteria for the participants:

*ADHD-only group*: 1) Aged 6-15 years; 2) educated in ordinary elementary or middle schools; 3) A diagnosis of ADHD confirmed through a clinical interview and a semi-structured interview by K-SADS-PL ().

*ADHD with ODD group*: 1) Aged 6-15 years; 2) educated in ordinary elementary or middle schools; 3) A diagnosis of ADHD comorbid ODD confirmed through a clinical interview and a semi-structured interview by K-SADS-PL.

*Control group***:** 1) Aged 6-15 years; 2) educated in ordinary elementary or middle schools; 3) no evidence of current or past major psychiatric disorders revealed in the K-SADS-PL assessment.

*Exclusion criteria that apply to all groups*: 1) a diagnosis of any psychiatric disorder other than ADHD or ODD; 2) history of head injury with loss of consciousness; 3) neurological abnormalities; 4) drug or substance abuse; 5) a full-scale IQ below 70. Additionally, of those who underwent MRI scans, any visible abnormalities (examined by an experienced radiologist, e.g., cyst) on the MRI images or non-right handedness also led to exclusion.

**Data Collection and Analysis**

1. Clinical Assessments: The parents were asked to score the Child Behavior Checklist (CBCL) with 0 = Not True, 1 = somewhat or sometimes true, and 2 = very true or often true. These items can be divided into eight factors, including withdrawn, somatic complaints, anxiety/depression, social problem, thought problem, attention problem, delinquent behavior, and aggressive behavior. CBCL was a valid tool. The reliability was 0.9, and its internal consistency ranged from 0.63 to 0.79 (10922023). Since the computer-based standardized T score is unavailable in the Chinese version (Biederman, Monuteaux, Kendrick, Klein, & Faraone, 2005), we used the original summing score of all items in each factor instead. The Behavior Rating Inventory of Executive Function (BRIEF) was used to assess everyday executive function in children. The Mandarin version was validated (Qian & Wang, 2007), with the reliability of all sub-scales ranging from 0.68 to 0.89 and the internal consistency ranging from 0.61 to 0.96 except for the initial subscale (17572784). It has 86 items, and each item was scored from 1 (none) to 3 (often), and these items provide eight non-overlapping factors reflecting commonly described domains of executive function, including inhibit, working memory, plan/organize, monitor, initiate, shift, organization of materials, and emotional control (Gioia et al., 2002). Again, the summing score of all items in each factor was utilized due to the absence of a standardized T score in the Mandarin version.

2. Brain Imaging protocols: 1) High-resolution 3D T1-weighted images were acquired using a magnetization-prepared rapid gradient echo (MPRAGE) sequence: 176 sagittal slices, TR/TE = 2530/2.25 ms, flip angle = 7°, FOV = 256 × 256 mm, 1.33 mm thickness with no gap, 1 × 1 mm in-panel resolution. 2) the fMRI scans were acquired using the following parameters: Single-shot echo-planar imaging (EPI) sequences were applied: TR = 2000 ms, TE = 30 ms, flip angle = 90°, thickness/skip = 3.5/0.7 mm, matrix = 64 × 64, field of view (FOV) = 200 × 200 mm, 33 axial slices, 240 volumes, 3 mm × 3 mm in-plane resolution; 3) the parameters for diffusion-weighted images: 49 axial slices, TR/TE=7200/104 ms, flip angle=90°, FOV=220×220 mm, in-plane resolution=1.7 × 1.7 mm, 2.5 mm thickness with no gap, 64 optimal nonlinear diffusion-weighted directions with b = 1000 s/mm2 and one image without diffusion weighting (i.e., b = 0 s/mm2);

3. preprocessing of resting-state function MRI: The fMRI and T1w images were preprocessed using FSL and Python. The following steps were applied sequentially: 1) remove the first ten time points; 2) realign to the middle volume; 3) grand mean scaling; 4) spatial smoothing at a Gaussian Kernel of 6 mm full-width at half-maximum; 5) To further correct for head motion artifact, the independent Components Analysis-based Automatic Removal Of Motion Artifacts (ICA-AROMA) technique was applied (Pruim et al., 2015). 6) remove signals of cerebrospinal fluid and white matter with nuisance regression; the white matter and cerebrospinal fluid masks were obtained based on T1w images and the FSL FAST algorithm; 7) high pass filtering (0.01 Hz); 8) the fMRI images were first registered to the subject-specific T1 images, and then to standard space. Individuals with excessive head motion (head motion >3 mm of translation or >3 degrees of rotation in any direction) were excluded. Seed-based functional connectivity was estimated using the subregions of the bilateral Amygdala as regions of interest (ROIs). The masks of the bilateral Amygdala were extracted from the Jülich histological atlas, resulting in 6 subregions, including the left and right cortical/medial (CM) parts, the left and right basolateral (BL) parts, and the left and right superficial (SF) parts. The spatial maps of these seed regions are presented in Figure. The timeseries of each subregion and the functional connectivity maps were built using dual regression.


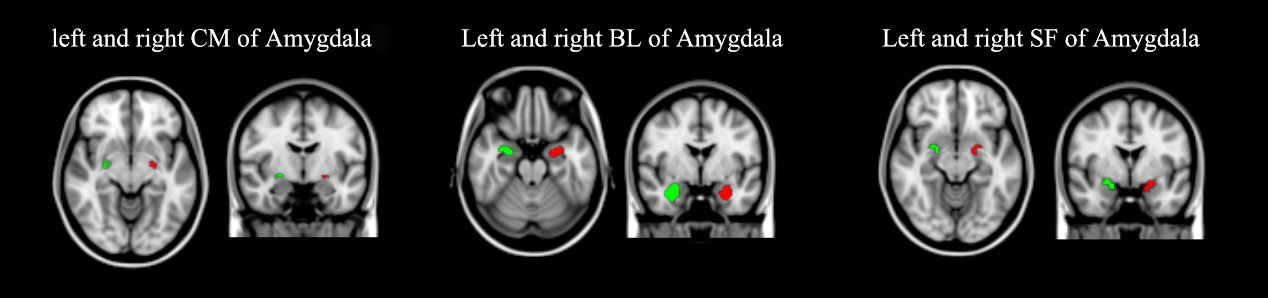


^sFigure 1: The spatial maps of the subregions of the bilateral Amygdala displayed on an MNI_T1_2mm template.^

4. preprocessing of diffusion-weighted images: All the images were preprocessed using FSL (https://fsl.fmrib.ox.ac.uk/fsl/docs/#/), using the following steps: 1) eddy current distortion; 2) motion parameters calculation; 3) Brain extraction. The resultant images were visually checked to ensure the quality of brain extraction. In addition, any individual showing excessive head motion, i.e., >3 mm of translation or >3 degrees of rotation in any direction, was excluded. The BedpostX and PROBTRACKX algorithm implanted in FSL was used to do the fiber tracking, producing “streamlines” that connect voxels between selected seed regions, e.g., subregions of the bilateral Amygdala, and target regions, e.g., the whole gray matter. The following parameters were utilized: streamline samples = 5000, step length = 0.5 mm, curvature threshold = 0.2.

**Supplementary Results**

sTable 1: Brain-behavior associations of those clusters showing significant group-wise differences in functional connectivity

|  | Withdrawn | Somatic Complaints | Anxiety/depression | Social problem | Thought problem | Attention problem | Delinquent behavior | Aggressive behavior |
| --- | --- | --- | --- | --- | --- | --- | --- | --- |
| L_CM_C1 | 1.84(0.25) | 3.83(0.0027) | 4.95(0.037) | 0.99(0.56) | 1.78(0.033) | 5.51(0.042) | 1.85(0.36) | 2.02(0.65) |
| L_CM_C2 | -0.34(0.80) | 0.94(0.38) | 1.58(0.42) | 0.56(0.70) | 0.30(0.67) | 2.30(0.31) | 0.66(0.69) | -1.13(0.77) |
| L_CM_C3 | 0.83(0.53) | 2.17(0.043) | 2.48(0.20) | 0.41(0.77) | 1.11(0.11) | 2.03(0.36) | 0.76(0.64) | -3.96(0.28) |
| L_CM_C4 | 1.67(0.33) | 3.77(0.0058) | 5.50(0.030) | 0.74(0.68) | 1.81(0.043) | 6.19(0.032) | 1.78(0.41) | 3.53(0.45) |
| R_CM_C1 | -0.36(0.75) | 0.77(0.41) | -0.71(0.67) | -1.66(0.16) | 0.39(0.51) | -2.41(0.22) | -1.23(0.39) | -2.36(0.46) |
| R_CM_C2 | 1.40(0.32) | 2.13(0.057) | 2.41(0.24) | -1.30(0.37) | 0.45(0.53) | -0.75(0.75) | 0.13(0.94) | 1.05(0.79) |
| R_CM_C3 | -0.91(0.58) | -0.95(0.47) | -1.43(0.55) | -3.24(0.058) | 1.38(0.10) | -2.39(0.38) | -2.54(0.20) | -7.68(0.09) |
| R_SF_C1 | -0.16(0.89) | 0.38(0.70) | -0.79(0.66) | -0.91(0.48) | 0.75(0.23) | 0.14(0.94) | -1.20(0.42) | 0.98(0.77) |
| R_SF_C2 | -0.50(0.63) | 0.55(0.49) | -0.90(0.54) | -0.43(0.69) | 0.44(0.41) | 0.55(0.75) | -1.47(0.23) | 0.023(0.99) |
| R_SF_C3 | 0.21(0.76) | 0.55(0.30) | -0.48(0.62) | 0.18(0.79) | 0.12(0.73) | 0.27(0.81) | -0.54(0.52) | 0.68(0.71) |
| R_SF_C4 | -0.039(0.98) | 0.069(0.97) | -2.36(0.40) | -2.68(0.17) | -2.06(0.036) | -0.73(0.82) | -2.08(0.37) | -0.68(0.90) |
| R_SF_C5 | 0.82(0.50) | 0.56(0.57) | 1.00(0.57) | -0.78(0.54) | 0.73(0.24) | 0.26(0.90) | -1.02(0.50) | 1.69(0.622) |
| R_SF_C6 | 2.05(0.42) | 0.63(0.75) | 2.51(0.49) | -0.88(0.74) | 0.88(0.50) | 6.79(0.10) | -0.75(0.81) | -4.46(0.53) |
| R_SF_C7 | 0.35(0.72) | 0.23(0.78) | 0.17(0.91) | -0.48(0.64) | 0.29(0.57) | 1.66(0.32) | -0.33(0.79) | 2.33(0.40) |

Abbreviations: L=Left; R=Right; CM=the cortical/medial (CM) part of the Amygdala; BL=the basolateral (BL) part of the Amygdala; SF=the superficial (SF) part of the Amygdala. C=Cluster. For instance, L_CM_C1 represents Cluster 1, showing group-wise differences in functional connectivity of the left cortical/medial (CM) part of the Amygdala. Details of these clusters can be found in Table 3 in the main text. * Indicates an FDR-corrected *P* value below 0.05.

sTable 2: Brain-behavior associations of those clusters showing significant group-wise differences in structural connectivity

|  | Withdrawn | Somatic Complaints | Anxiety/depression | Social problem | Thought problem | Attention problem | Delinquent behavior | Aggressive behavior |
| --- | --- | --- | --- | --- | --- | --- | --- | --- |
| L_SF_SC1 | 0.027(0.0043)* | 0.013(0.097) | 0.047(0.00040)* | 0.011(0.26) | 0.012(0.010)* | 0.010(0.052) | 0.025(0.024)* | 0.044(0.085) |
| L_SF_SC2 | 0.070(0.75) | 0.13(0.46) | 0.32(0.30) | -0.30(0.18) | 0.0076(0.95) | -0.73(0.039) | 0.30(0.23) | -0.19(0.75) |
| L_SF_SC3 | 0.013(0.13) | 0.0058(0.39) | 0.027(0.025) | 0.0055(0.51) | 0.0036(0.38) | -0.0047(0.73) | 0.0095(0.33) | 0.026(0.25) |
| L_SF_SC4 | 0.074(0.19) | 0.11(0.026) | 0.16(0.050) | 0.086(0.14) | 0.092(0.0010)* | 0.049(0.62) | 0.21(0.0021)* | 0.36(0.021) |

Abbreviations: L=Left; R=Right; CM=the cortical/medial (CM) part of the Amygdala; BL=the basolateral (BL) part of the Amygdala; SF=the superficial (SF) part of the Amygdala. SC=Cluster. For instance, L_SF_SC1 represents Cluster 1, showing group-wise differences in structural connectivity of the left superficial (SF) part of the Amygdala. Details of these clusters are in Table 3 in the main text. * Indicates an FDR-corrected *P* value below 0.05.

sTable 3: Brain-behavior associations of those clusters showing significant group-wise differences in functional connectivity

|  | Inhibit | Shift | Emotional control | Initiate | Working Memory | Planning | Organization of material | Monitor |
| --- | --- | --- | --- | --- | --- | --- | --- | --- |
| L_CM_C1 | 5.74(0.063) | 5.32(0.0023) * | 3.34(0.17) | 3.43(0.14) | 9.92(0.0049) * | 6.43(0.099) | 3.29(0.18) | 6.63(0.013) |
| L_CM_C2 | -0.23(0.93) | 1.04(0.50) | -1.87(0.38) | -1.40(0.50) | -0.66(0.83) | -4.63(0.17) | -0.036(0.99) | -1.56(0.50) |
| L_CM_C3 | 1.40(0.58) | 3.81(0.0064) * | 0.76(0.71) | 1.10(0.58) | 3.63(0.22) | 1.78(0.58) | 0.70(0.72) | 0.14(0.95) |
| L_CM_C4 | 7.10(0.026) | 5.59/0.0020) * | 4.77(0.061) | 3.85(0.12) | 10.94(0.0027) * | 7.83(0.052) | 4.25(0.091) | 7.34(0.0080) * |
| R_CM_C1 | -3.16(0.15) | 0.43(0.73) | -2.29(0.20) | -1.45(0.40) | -0.23(0.93) | -2.92(0.30) | 0.091(0.96) | -1.69(0.38) |
| R_CM_C2 | -0.092(0.97) | 0.10(0.95) | -1.82(0.41) | -1.86(0.39) | 1.92(0.55) | -2.32(0.51) | -0.40(0.85) | -1.1(0.64) |
| R_CM_C3 | 2.33(0.44) | 2.26(0.19) | -0.37(0.88) | -0.43(0.86) | 1.94(0.60) | 1.48(0.71) | 2.66(0.27) | 2.03(0.47) |
| R_SF_C1 | 1.94(0.37) | 1.51(0.22) | 1.04(0.56) | 0.74(0.67) | 3.06(0.24) | 3.21(0.26) | 1.66(0.35) | 3.24(0.099) |
| R_SF_C2 | 3.21(0.13) | 2.29(0.060) | 3.75(0.032) | 2.01(0.23) | 3.63(0.16) | 3.08(0.26) | 1.21(0.47) | 1.47(0.44) |
| R_SF_C3 | -1.32(0.32) | 0.53(0.48) | -0.66(0.54) | 0.11(0.92) | -0.34(0.83) | -0.19(0.91) | 0.28(0.78) | -0.43(0.72) |
| R_SF_C4 | 0.82(0.82) | 2.23(0.26) | -0.72(0.80) | 0.49(0.86) | 5.50(0.18) | 6.34(0.16) | 2.50(0.35) | 2.71(0.38) |
| R_SF_C5 | 0.14(0.95) | -0.010(0.99) | 1.10(0.57) | -2.13(0.25) | 0.90(0.74) | 0.84(0.78) | 1.27(0.49) | 1.20(0.57) |
| R_SF_C6 | 0.080(0.99) | 4.69(0.13) | -3.62(0.41) | 5.78(0.18) | 6.03(0.36) | 7.21(0.30) | 7.79(0.075) | 3.77(0.44) |
| R_SF_C7 | 0.025(0.99) | 0.078(0.94) | -0.15(0.92) | 0.88(0.52) | 2.54(0.21) | 1.77(0.43) | 1.44(0.29) | 1.98(0.20) |

Abbreviations: L=Left; R=Right; CM=the cortical/medial (CM) part of the Amygdala; BL=the basolateral (BL) part of the Amygdala; SF=the superficial (SF) part of the Amygdala. C=Cluster. For instance, L_CM_C1 represents Cluster 1, showing group-wise differences in functional connectivity of the left cortical/medial (CM) part of the Amygdala. Details of these clusters are in Table 3 in the main text. * Indicates an FDR-corrected *P* value below 0.05.

sTable 4: Brain-behavior associations of those clusters showing significant group-wise differences in structural connectivity

|  | Inhibit | Shift | Emotional control | Initiate | Working Memory | Planning | Organization of material | Monitor |
| --- | --- | --- | --- | --- | --- | --- | --- | --- |
| L_SF_SC1 | 0.018(0.40) | 0.012(0.35) | 0.0062(0.72) | 0.0048(0.79) | 0.013(0.61) | 0.016(0.59) | 0.013(0.43) | 0.0075(0.70) |
| L_SF_SC2 | -0.59(0.18) | -0.096 (0.70) | -0.032(0.93) | -0.79(0.018) | -1.08(0.038) | -1.32(0.023) | -0.41(0.23) | -0.51(0.22) |
| L_SF_SC3 | 0.0050 (0.77) | -0.0020(0.84) | 0.0048(0.73) | -0.0019(0.89) | 0.00045(0.98) | 0.019(0.40) | 0.017(0.18) | 0.010(0.48) |
| L_SF_SC4 | 0.26(0.019) | 0.033(0.56) | 0.22(0.0050) | 0.11(0.19) | 0.14(0.29) | 0.20(0.13) | 0.15(0.054) | 0.24(0.015) |

Abbreviations: L=Left; R=Right; CM=the cortical/medial (CM) part of the Amygdala; BL=the basolateral (BL) part of the Amygdala; SF=the superficial (SF) part of the Amygdala. SC=Cluster. For instance, L_SF_SC1 represents Cluster 1, showing group-wise differences in structural connectivity of the left superficial (SF) part of the Amygdala. Details of these clusters are in Table 3 in the main text. * Indicates an FDR-corrected *P* value below 0.05.
